# Shape-Persistent Dendrimers

**DOI:** 10.3390/molecules28145546

**Published:** 2023-07-20

**Authors:** Yao-Chih Lu, Roberto Anedda, Long-Li Lai

**Affiliations:** 1 Department of Applied Chemistry, National Chi Nan University, Puli 545, Taiwan; yaochihlu@gmail.com; 2 Porto Conte Ricerche Srl, S.P. 55 Porto Conte-Capo Caccia, Km 8,400, Loc. Tramariglio 15, 07041 Alghero, Italy

**Keywords:** dendrimer, shape-persistent, void space, sensing, adsorbing

## Abstract

Dendrimers have a diverse and versatile morphology, frequently consisting of core, linking, and peripheral moieties. Dendrimers with flexible linkers, such as PAMAM, cannot retain the persistent shape of molecules, and this has been widely explored and reviewed previously; nevertheless, dendrimers with stiff linkers can preserve the persistent shape of the dendrimers, which has been reported considerably less. This review thus focuses on addressing shape-persistent dendrimers with rigid linking moieties discovered in recent years, i.e., from 2012 to 2023. Shape-persistent dendrimers with an interstitial gap between the dendritic frames in the solid state may or may not let the intramolecular void space be accessible for guest molecules, which largely depends on whether their peripheral groups are flexible or non-flexible. In this paper, eight articles on shape-persistent dendrimers with a flexible alkyl periphery, which may exhibit mesogenic phases upon thermal treatment, and eight articles on shape-persistent dendrimers with a non-flexible periphery, which may allow external ions, gases, or volatile organic compounds to access the interstitial gaps between dendritic frames, are reviewed.

## 1. Introduction

Dendrimers are usually composed of a combination of three constructing moieties, i.e., central cores, linking bridges, and peripheral groups, and since the first discovery of dendrimers in the early 1970s [1], their synthesis and the study of their related physical properties have been ongoing. Dendrimers can be prepared in a controllable way, using convergent, divergent, and combined processes, and thus have a predictable morphology, which gives them an advantage over polymers [2]. Other characteristics of dendrimers include their good solubility in organic solvents and the mono-dispersity of their molecular weights, which allow this kind of macromolecule to be easily purified and re-processed after their usage. In particular, dendrimers can have a two-dimensional or three-dimensional orientation, thus inducing the internal void inside the dendritic framework. Therefore, they have been reported to adsorb metal ions or small molecules in solution for use in drug delivery [3,4,5,6,7,8,9] and catalysis [10,11,12,13,14,15,16]. In addition to central cores and peripheral functionalities, these types of dendrimers usually consist of flexible linkers, and in this case, their persistent molecular conformation is not easy to maintain and the intramolecular pores are not accessible to guest molecules in the solid state. This kind of dendritic molecule may have PPI (polypropylimine), PAMAM (polyamidoamine), Si derivatives (carbosilanes, siloxanes, and carbosilazanes), polyether, and polyester networks. Rigid or mesogenic peripheral groups have been found to be attached to the flexible linkers, and corresponding dendrimers have been broadly investigated for applications in the fields of light harvesting and liquid crystals. These dendrimers have already been reviewed extensively [2,17,18,19,20,21,22], and will not be included in this article.

To keep the void space in the dendritic framework in the solid state accessible to external molecules, as in metal–organic frameworks (MOFs) [23,24,25], covalent–organic frameworks (COFs) [26,27,28], or covalent–organic polymers (COPs) [29,30,31], dendrimers must keep rigid linkers to maintain their dendritic shapes. However, the rigid linkers always make the dendrimers relatively insoluble in organic solvents, which in turn leads to difficult preparation and purification processes. For example, a shape-persistent dendrimer based on a triazine unit was prepared by Takagi in 2000 [32], and, probably due to its low solubility in organic solvents and difficult purification, no follow-up studies were further reported. To solve the difficulty of low solubility, Simanek introduced flexible linkers and flexible or bulky peripheral groups to construct related dendrimers based on a triazine moiety, which were then used as drug-transporting templates in the corresponding studies [33]. Shape-persistent dendrimers, similarly to other porous materials, may have void spaces in the solid state, which may or may not be accessible to external molecules, mostly depending on the flexible or non-flexible nature of their peripheral groups. Furthermore, they may have the characteristics of good solubility in organic solvents and ease of reprocessing after use, which is almost impossible to achieve for MOFs, COFs, and COPs; therefore, although their synthesis and purification are still fraught with challenges, some related work has been explored. Shape-persistent dendrimers containing flexible peripheral groups may or may not allow their intramolecular void spaces to be accessible to gases in the solid state, and those with a non-flexible periphery may allow the interstitial gaps between dendritic frames to be accessible to external ions, gases, or volatile organic compounds. Because not many shape-persistent dendrimers have been explored and investigated, and most of them were extensively reviewed in 2016 and 2018 by Mullen and Hammer et al. [34,35], this review only covers shape-persistent dendrimers with rigid linkers discovered in recent years, i.e., from 2012 to 2023, excluding the related works of Mullen and Hammer et al. The reviewed dendrimers are categorized into (1) 2D molecules with co-planar rigid linkers and flexible peripheral groups, (2) 2D molecules with co-planar rigid linkers and bulky peripheral groups, (3) 2D or 3D molecules with highly twisted rigid linkers and (4) 3D molecules with three-dimensional rigid linkers or cores. Interestingly, 2D dendrimers with coplanar rigid linkers and flexible peripheral groups can appear as disc-shaped molecules and exhibit columnar mesogenic phases upon thermal treatment. The 2D dendrimers with coplanar rigid linkers and bulky peripheral groups and 3D dendrimers may contain a void space in their solid states for adsorbing gases or volatile organic compounds.

## 2. 2D Shape-Persistent Dendrimers with Co-Planar Rigid Linkers and Flexible Peripheral Groups

**Figure 1:**
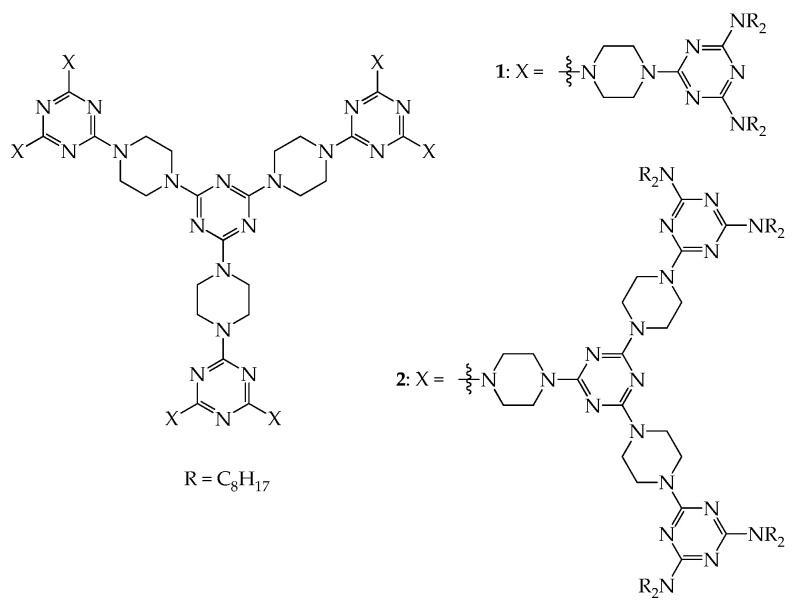
Structures of dendrimers **1** and **2**.

**Figure 2:**
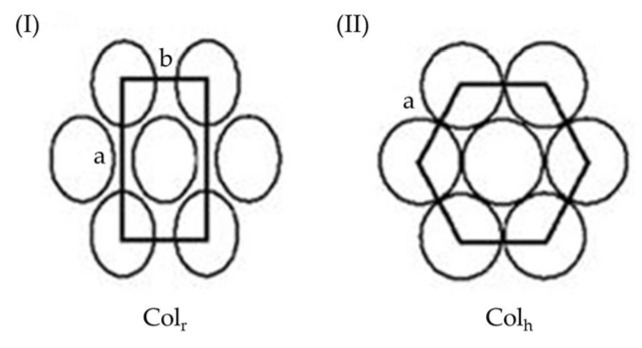
Schematic representation of (**I**): the 2D lattices of columnar rectangular (Col_r_) and (**II**): columnar hexagonal (Col_h_) phases; a and b indicate the distances between column discs.

As indicated earlier, shape-persistent dendrimers generally possess rigid linkers in the dendritic frame, and thus their solubility in solvents may become an issue during their purification. To avoid such concerns, flexible groups could be attached at their periphery, and to this end, the convergent approach has been generally used as a synthetic strategy. In 2012, Lai and co-workers prepared two shape-persistent dendrimers using piperazine and 1,3,5-triazine as rigid linkers and NR_2_ (R = C_8_H_17_) as flexible peripheral groups, as shown in Figure 1 [36]. These two dendrimers contain a three-fold symmetry at a molecular level and thus self-assemble into liquid crystals upon thermal treatment (Table S1). Based on powder-XRD studies, both dendrimers were observed to have 2D rectangular packing in their mesogenic state (Figure 2I); their lattice constants *a* and *b* were calculated to be 57.10 and 41.52 Å, respectively, for dendrimer **1** and 64.99 and 60.16 Å, respectively, for dendrimer **2**. The confirmation of the rectangular columnar LC phase suggests that dendrimers **1** and **2** have a rigid 2D and planar dendritic frame like a disk plate and are thus able to self-assemble upon thermal treatment to form the columnar phase. Based on the schematic representation of the 2D lattices of columnar rectangular stacking (Figure 2I) [37], it may be difficult to estimate the average diameter of disk-like molecules because they may have an ellipse shape in the mesogenic state. However, it is reasonable to assume that the average diameter of dendrimer **2** should be greater than that of **1** because dendrimer **2** contains more constructing linkers (piperazine and 1,3,5-triazine) than dendrimer **1**.

**Figure 3:**
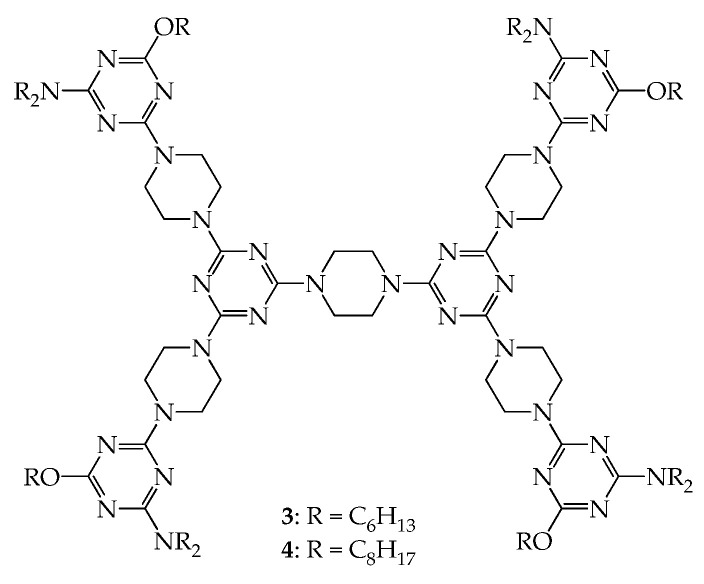
Structures of dendrimers **3** and **4**.

Dendrimers **3** and **4**, as shown in Figure 3, have the same rigid linkers as those of dendrimers **1** and **2** but different flexible groups, i.e., NR_2_ and OR (R = C_8_H_17_, C_6_H_13_), at their periphery [38]. Both dendrimers also exhibited mesogenic properties upon thermal treatment (Table S1). As observed from powder-XRD investigation, dendrimers **3** and **4** were also observed to have a 2D disk-like frame. However, dendrimer **4** is arranged in hexagonal packing in the mesogenic state, and its lattice constant *a* was calculated to be 36.0 Å; dendrimer **3** is arranged in rectangular packing with the lattice constants *a* = 51.5 Å and *b* = 20.2 Å. As shown in Figure 2II, the two column discs along axis *a* in the hexagonal packing in the mesogenic state are close to each other [37]. Therefore, it is reasonable to estimate, from the packing of disk-like dendrimer **4**, that the average diameter of column discs is ~36.0 Å.

**Figure 4:**
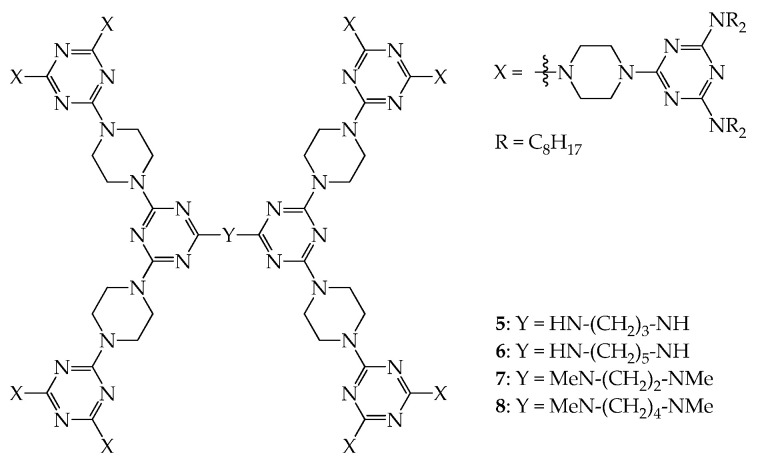
Structures of dendrimers **5**, **6**, **7**, and **8**.

Dendrimers **1**–**4** all have rigid cores, linkers, and flexible peripheral groups and thus behave as disk-like molecules. It has been observed that dendrimers with flexible cores, rigid linkers, and flexible peripheral groups also behave accordingly. Dendrimers **5**–**8**, as shown in Figure 4, were prepared using a convergent approach, and all exhibit mesogenic phases in the thermal process [39,40] (Table S1). The powder-XRD studies also confirmed that dendrimers **5**–**8** all have a 2D disk-like frame. Dendrimers **5**, **7**, and **8** all have hexagonal packing, and their lattice constant *a* is estimated to be 38.2, 40.8, and 38.6 Å, respectively; dendrimer **6** shows rectangular packing in the mesogenic state with the lattice constants *a* = 57.1 Å and *b* = 41.5 Å. Based on a 2D lattice of columnar hexagonal stacking in the mesogenic state, as demonstrated in Figure 2II [37], the average diameter of the column disc for dendrimer **5** is ~38.2 Å, which is slightly less than that of dendrimer **8** (38.6 Å), due to the shorter length of the core in **5**. However, the length of the core in dendrimer **7** is shorter than that in dendrimers **5** and **8**, but the average diameter of column disc **7** is ~40.8 Å, which is much greater than that of dendrimers **5** and **8**. According to the literature [40], the molecular volume of dendrimer **7** (~10,533 Å^3^) is similar to that of **8** (~10,688 Å^3^) because the molecular weight of **7** (6034 Å^3^) is similar to that of **8** (6062 Å^3^). However, the cell volume of **7** (~12,979 Å^3^) is much greater than that of **8** (~11,628 Å^3^), meaning that the steric congestion of the core in dendrimer **7** can cause the disk-shaped dendrimers to rearrange into a minor order. In other words, the mutual gliding of dendritic molecules within the same columns may arise due to steric congestion of the central core in dendrimer **7** and thus increase the mean diameter of the column disk in the hexagonal packing.

**Figure 5:**
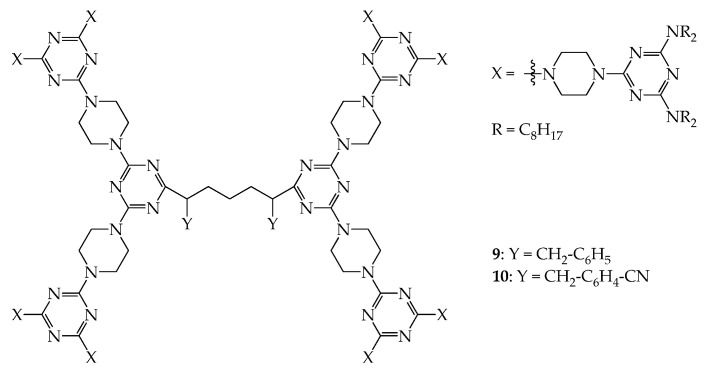
Structures of dendrimers **9** and **10**.

Dendrimers **9** and **10**, as demonstrated in Figure 5, were prepared, and their physical properties were then investigated, further confirming that the steric congestion of dendritic cores may affect the average size of the disc column of the dendrimer in the mesogenic state [41]. The related mesogenic ranges are provided in Table S1. The XRD reflection pattern also indicates that dendrimer **9** has a hexagonal columnar phase with a calculated lattice constant of 41.9 Å, which is much longer than that of dendrimer **5** (~38.2 Å). Both dendrimers **5** and **9** have NY-CH_2_-CH_2_-CH_2_-NY as their central linkers (**5**: Y = H and **9**: Y = CH_2_-C_6_H_5_), but the steric congestion in dendrimer **9** is more significant than that in **5**. This is related to the two phenylmethyl substituents on the nitrogen atoms, which affect the morphology of dendrimer **9**, resulting in the mutual gliding of dendritic molecules within the same columns. Therefore, the cell volume of **9**, ~13,686 Å^3^, is much greater than that of **5** (~11,230 Å^3^), although the molecular volumes of dendrimers **9** and **5** are not much different (~10,981 and 10,662 Å^3^, respectively) because of their similar molecular weights (**9**: 6202 and **5**: 6022). Very interestingly, dendrimer **10**, with the central linker NY-CH_2_-CH_2_-CH_2_-NY (Y = CH_2_-C_6_H_4_-CN), also has a similar XRD pattern to **9** in the mesogenic state, and the corresponding lattice constant was calculated to be 38.4 Å, which is similar to that of dendrimer **5** but much less than that of **9**. The CH_2_-C_6_H_4_-CN substituent in the core of dendrimer **10** does not seem to significantly enhance the mutual gliding of dendritic molecules within the same columns, as demonstrated in **9**, thus enlarging the average diameter of the column disc. The molecular volume of **10** was estimated to be ~11,226 Å^3^ based on the powder-XRD data, which is slightly greater than those of **5** (~10,662 Å^3^) and **9** (~10,981Å^3^) because the molecular weight of **10** (6252) is slightly greater than that of **5** (6022) and **9** (6202). The cell volume of **10** was calculated to be 11,510 Å^3^, which is, however, close to that of dendrimer **5** (~11,230 Å^3^) but much less than that of **9** (~13,686 Å^3^). According to the literature [41], the distance between dendritic frames within the column in **10** is ~13.68 Å, which is slightly larger than that of dendrimers **5** and **9**, which demonstrate distances of 13.14 and 13.28 Å, respectively. Based on all these results, it may be concluded that the CN dipole in the central core of the dendrimer increases the distance between dendritic frames within the column and this, to some extent, reduces the steric congestion by the CH_2_-C_6_H_4_-CN moiety, thus resulting in negligible mutual gliding between dendritic molecules.

**Figure 6:**
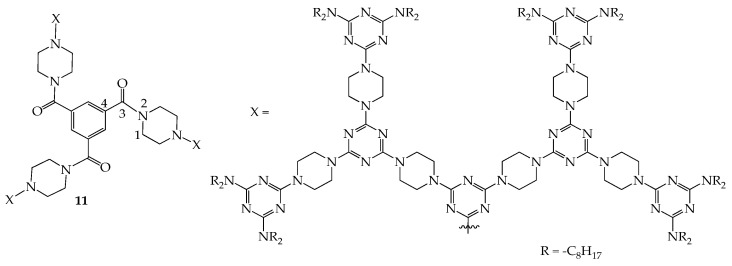
Structures of dendrimer **11**.

Although dendrimers with rigid linkers and flexible peripheral groups can exhibit a persistent shape, the inner void space inside the dendritic framework is rarely reported to be accessible to external gases in the mesogenic or solid state. Very recently, a dendrimer with a triamidobenzene core, as demonstrated in Figure 6, was synthesized, and it was observed that it can adsorb Xe gas in the mesogenic and solid states based on a ^129^Xe-NMR spectroscopy study at various temperatures [42]. The related mesogenic ranges are provided in Table S1. Based on the XRD data, dendrimer **11**, with 2D packing, shows a rectangular columnar phase with the lattice constants of *a* = 57.5 Å and *b* = 41.9 Å. As shown in the literature [42], the dihedral angle of C1-N2-C3-C4 of **11** in the gas phase was calculated to be 25.0 °C, which forces the central core of dendrimer **11** to be non-coplanar, making the inner pore inside the dendritic framework available for guest gases in the mesogenic or solid state. The NMR study revealed that ^129^Xe gas can be absorbed into the free void space inside the dendrimer at a temperature range of about −20~80 °C upon heating and at a range of about 105~8–35 °C upon cooling. According to the literature [42], this is the first report demonstrating that a liquid crystalline dendrimer possesses a free void space in the mesogenic and solid states.

**Figure 7:**
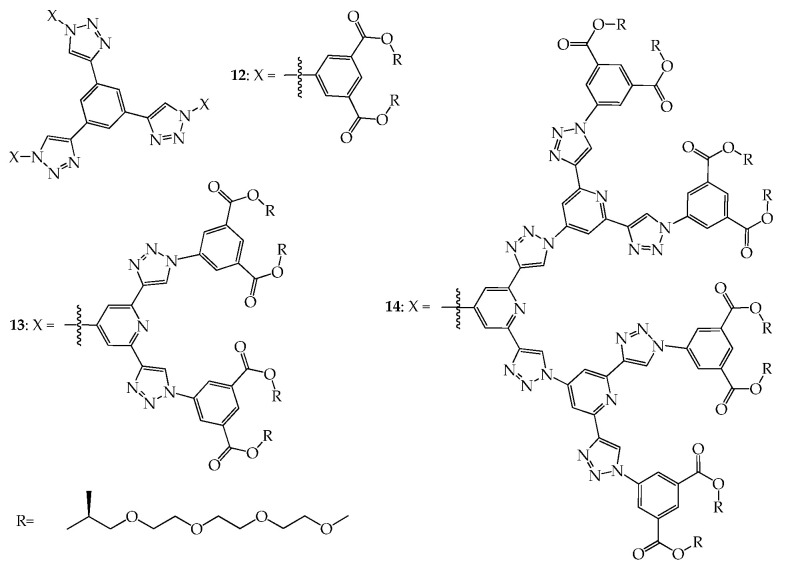
Structures of dendrimers **12**–**14**.

Using a convergent and click approach, shape-persistent dendrimers **12**–**14**, as shown in Figure 7, were prepared by Malah et al. [43]. These amphiphilic dendrimers with a disc shape assemble in water to produce cylindrical assemblies that undergo a thermosensitive phase transition from a nanorod to columnar phase. The structure of dendrimer arrays in solution was investigated via small-angle X-ray scattering (SAXS), and the length and radius of the nanorods were measured; the radius of **12**, **13**, and **14** is about 1.41, 1.86, and 2.31 nm, respectively, and the length of **12**, **13**, and **14** is about 14.7, 16.8, and 57.7 nm, respectively. Since lengths reflect the number of stacked dendrimer discs in cylindrical columns, it could be concluded that the stacked numbers increase with generation number, being 42, 48, and 165 for **12**, **13**, and **14**, respectively. The use of click approaches for preparing shape-persistent dendrimers was also reported in 2010 by Kakkar [44] and in 2014 by Mullen [45], respectively. The work of Kakkar also involved flexible peripheral groups similar to the dendritic structures investigated by Malah, and thus will only be briefly mentioned here. As indicated, the work of Mullen before 2018 has been already reviewed [34,35] and thus will not be described here in detail.

**Figure 8:**
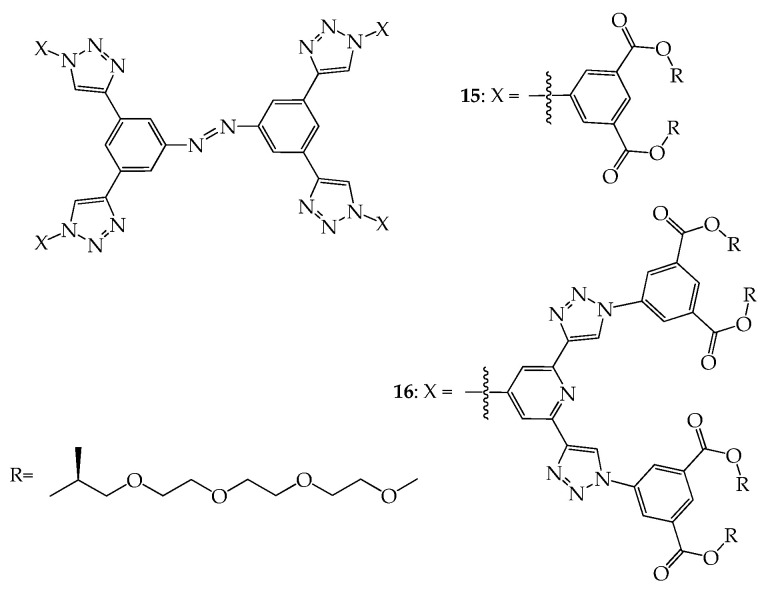
Structures of dendrimers **15** and **16**.

Malah et al. further prepared dendrimers **15** and **16** with azobenzene as the central core and long-chain ether branches as peripheral groups (Figure 8), which were observed to self-assemble into highly ordered and uniform nanofibers [46]. The aggregated fibers, characterized by TEM and SEM microscopies, were observed to be uniform with an average diameter of 155.6–250.3 nm, likely resulting from π-stacking of the aromatic core units and the interaction between alkyl chains.

## 3. 2D Shape-Persistent Dendrimers with Co-Planar Rigid Linkers and Bulky Peripheral Groups

**Figure 9:**
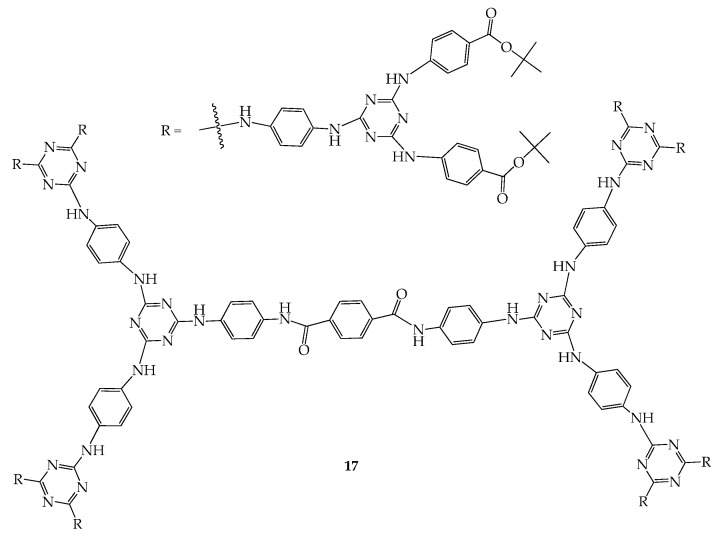
Structures of dendrimer **17**.

Instead of using flexible groups, the problem of solubility in organic solvents for shape-persistent dendrimers may be overcome by introducing a bulky functionality at their periphery. Dendrimer **17,** which demonstrated good solubility in THF, was successfully prepared and observed to adsorb CO_2_ in the solid state. Based on a CO_2_ isotherm at 195 K, the BET (Brunauer–Emmett–Teller) and Langmuir surface areas of **17** were estimated to be 154.32 and 292.92 m^2^·g^−1^, respectively (Figure 9). Interestingly, dendrimer **17** exhibits low adsorption of N_2_ gas, and this is the first time that dendrimers have been reported to exhibit superior selective adsorption of CO_2_ over N_2_ in the literature [47]. Details of a typical process used for determining the free void spaces of samples have been added as Supplementary Materials.

**Figure 10:**
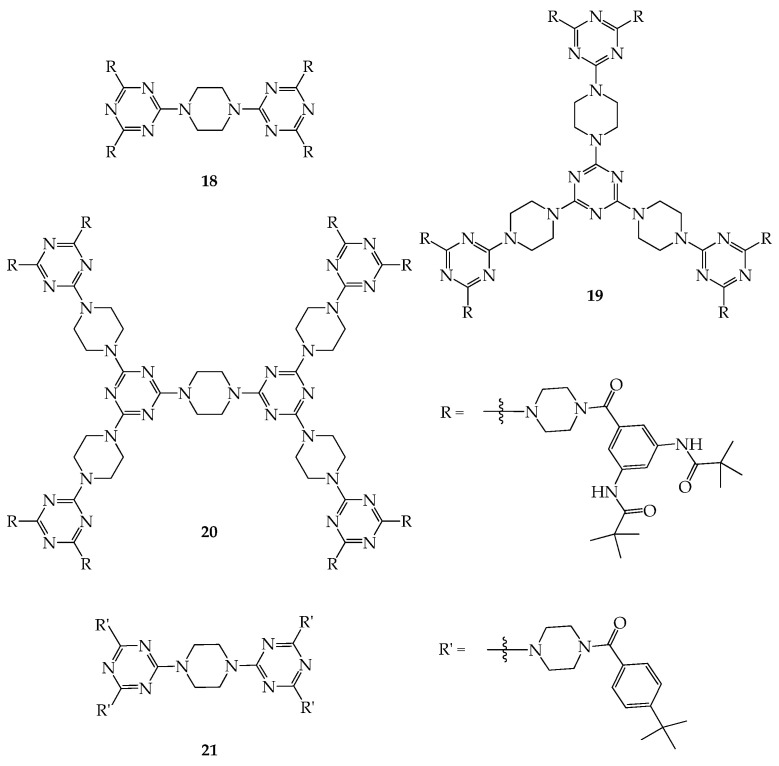
Structures of dendrimers **18**–**21**.

Dendrimers **18**–**20** were further prepared and investigated for their porous properties (Figure 10). As shown in the literature [48], the BET surface areas of **18**–**20** with the piperazine and triazine linkers are similar to each other despite their various molecular weights (ranging from 1791 to 2890) and are in the range of 136–138 m^2^/g based on the CO_2_ isotherms of dendrimer **18**–**20** at 195 K. Because of the strong H-bond interaction between the peripheral R groups of **18**–**20**, the triamidobenzene moiety (TAB) may not imbed in the interstitial space of the bulk dendrimers in the solid state, and therefore the BETs of **13**–**15** are similar to each other and majorly depend on the void spaces constructed by the piperazine and triazine moieties. This assumption was further supported by the BET value of dendrimer **21** (~47 m^2^/g). This is due to the absence of H-bonding interactions between the peripheral groups of dendrimer **21** (Figure 10). Interestingly, the BETs of **18**–**20** were observed to be slightly lower than that of a COF (~165 m^2^/g) prepared from the reaction of piperazine with triazine [49], which is reasonable because some of the void space in **18**–**20** is occupied by the TAB groups inside the dendritic framework.

**Figure 11:**
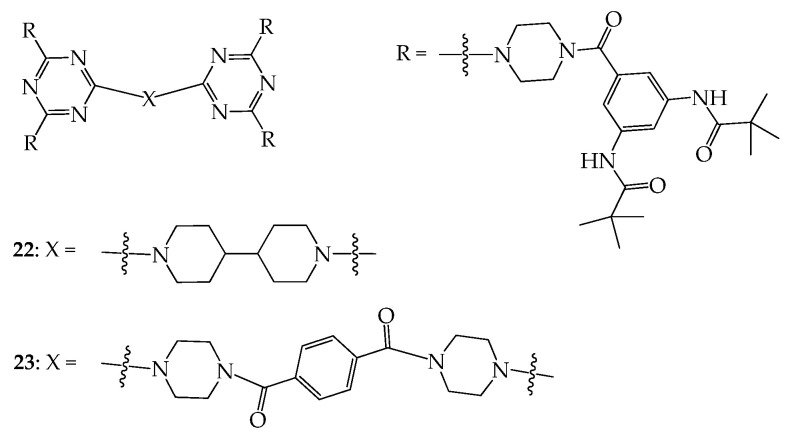
Structures of dendrimers **22** and **23**.

Based on the peripheral triamidobenzene moiety (TAB), dendrimers **22** and **23** were further prepared, and their void spaces were then studied (Figure 11). The BET surface areas of **22** and **23**, on the basis of the CO_2_ isotherm at 195 K, were calculated to be 191.8 and 212.3 m^2^/g, respectively, which are correspondingly 41 and 56% higher than that of **18** (136.0 m^2^/g) [50]. As shown in the literature [48], the TAB group does not imbed in the dendritic framework in the solid state, so the surface areas of **22** and **23** should be greater due to the longer chain length of their central cores. Dendrimers **22** and **23** were further used for adsorbing pyridines, and it was demonstrated that one molecule of **22** and **23** can adsorb 7 and 24 equivalents of pyridine, respectively. According to the literature, the adsorption capacity of **23** in pyridine is equivalent to 946.2 mg/g, which is the best value reported so far, being over twice the amount (400.8 mg/g) reported in the literature [51].

## 4. 2D or 3D Shape-Persistent Dendrimers with Highly Twisted Rigid Linkers

**Figure 12:**
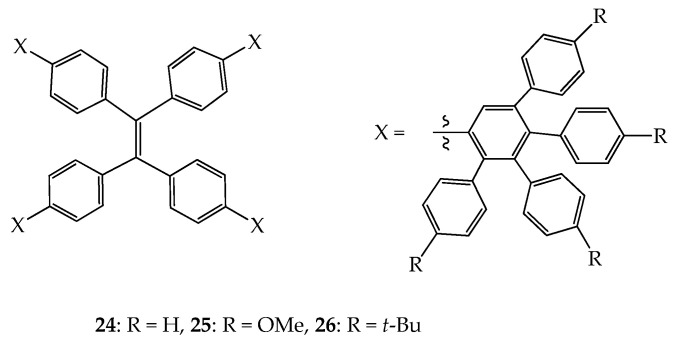
Structures of dendrimers **24**–**26**.

Another approach to increasing the solubility of shape-persistent dendrimers in organic solvents is to introduce highly twisted moieties such as polyphenylenes as rigid linkers. Based on this strategy, a few porous shape-persistent dendrimers have been prepared; among them, several well-known 2D or 3D dendrimers with rigid polyphenylene linkers have been prepared and studied by Mullen and Hammer et al. These polyphenylene dendrimers can be used as sensors for volatile organic compounds or nanocarriers for small molecules, which have been extensively reviewed in 2018 [34] and 2016 [35], and therefore, the related works before 2018 will not be further addressed in this article. Instead of sensing and delivering molecules, dendrimers **24**–**26**, which are based on polyphenylene linkers, as shown in Figure 12, have been recently studied regarding their emission variation in response to the stimuli of external light, mechanical force, and volatile organic vapors. Due to the steric effect on the peripheral group, which leads to various kinds of packing in the solid state, the sensitivity of emission variation, therefore, is significantly dependent on the external stimuli [52].

## 5. 3D Molecules with Three-Dimensional Rigid Linkers or Cores

**Figure 13:**
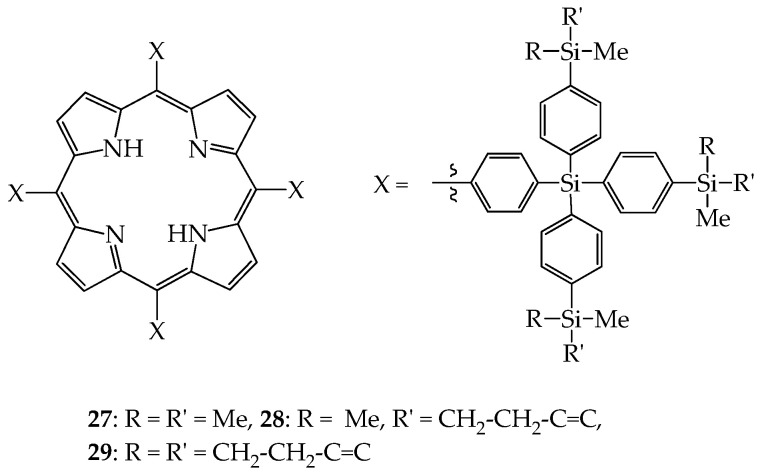
Structures of dendrimers **27**–**29**.

Three-dimensional rigid linkers or central cores can also be incorporated to increase the solubility of shape-persistent dendrimers in organic solvents. A 3D rigid linker of a silane derivative was used to prepare dendrimers **27**–**29** with porphyrin as a central core (Figure 13). Because of the introduction of a tetraphenylsilane linker, dendrimers **27**–**29** can be purified efficiently. However, only a slight bathochromic shift in the Soret and Q bands of the porphyrins was observed in the UV-Vis spectra compared to the non-dendritic porphyrins [53].

**Figure 14:**
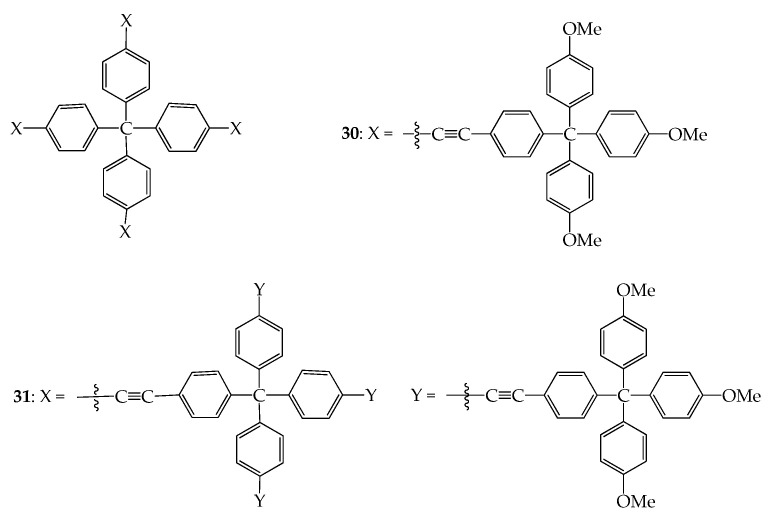
Structures of dendrimers **30** and **31**.

The shape-persistent dendrimers containing a 3D central core may be more effective in maintaining void space when compared with those with 3D rigid linkers. This strategy was reported previously by Ma and Pei et al. [54]. In a similar approach, Torneiro et al. prepared dendrimers **30** and **31** using a tetraphenylmethane unit as the dendritic core (Figure 14) [55]. Such structures with tetraphenylmethane units at their core and nodes are connected by rigid rod-like ethynylene linkers in a convergent synthetic approach. Alternatively, a divergent synthetic route has also been proposed by Torneiro et al. [56]. The first generation of the dendrimer has a star-like shape with a diameter of 3 nm, whereas the second generation shows a branched, porous, and well-defined globular structure with a hydrophobic interior. The second generation of dendrimer has a very narrow dimensional distribution, with a hydrodynamic radius of about 2.3 nm, as measured via dynamic light scattering. Both dendrimers, especially the second generation, showed exceptional fluorescent properties, with a quantum yield at room temperature of 65% and a very narrow emission band.

**Figure 15:**
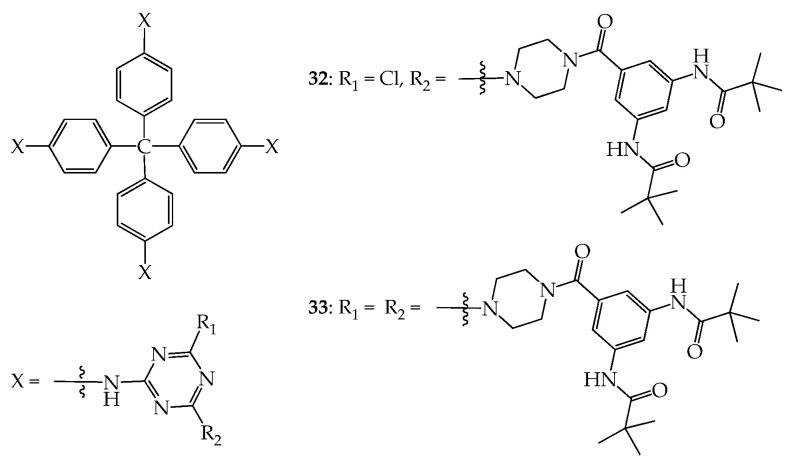
Structures of dendrimers **32** and **33**.

Dendrimers **32** and **33** with a tetraphenylmethane moiety as a dendritic core were also prepared by Lai et al to study their void space (Figure 15). The BET surface areas of **32** and **33** based on the CO_2_ isotherm at 195 K were calculated to be 177.6 and 213.7 m^2^/g, respectively [57]. The void space of **32**, as studied by means of CO_2_ absorption, is slightly less than that of **33**, and therefore, one molecule of **32** and **33** can adsorb 1.37 and 1.80 equivalents of toluene, respectively, which is rather consistent with the void space inside their dendritic framework. However, one molecule of **32** and **33** can adsorb 4.07 and 16.07 equivalents of benzonitrile, respectively, which is very surprising. This suggests that when more and more benzonitrile is adsorbed, the frameworks of dendrimers **32** and **33** progressively expand. Thus, the frameworks of **32** and **33** can be regarded as flexible. Because dendrimer **33** contains more TAB moieties at the periphery than dendrimer **32**, the framework of **33** provides more H-bond interaction sites for guest molecules than dendrimer **32**, allowing more benzonitrile to be adsorbed correspondingly.

## 6. Possible Applications

Although 2D or 3D shape-persistent dendrimers may contain void space inside their frameworks because of their rigid linkers, this can often lead these molecules to have low solubility in organic solvents. To solve this problem for easy purification during their preparation or reprocessing after use, 2D rigid dendrimers are usually constructed with flexible alkyl chains at their periphery, thus yielding mesogenic phases upon heat treatment, as shown previously. Because their liquid crystalline properties allow shape-persistent dendrimers to possess a good self-assembling ability over a long range, these dendrimers have more uniform stackings in the mesogenic states and possess potential applications for opto-electronic devices [58,59,60,61]. For example, carbon nanotubes or dendritic emitters can be uniformly dispersed in liquid crystalline materials, thus reducing the quenching effect in an organic light-emitting diode device [58,59]. Mesogenic dendrimers with an aromatic core, such as the structures **12**–**14** as cores and linkers, may show good charge mobility in related devices, such as organic field-effect transistors, because of their proper arrangement, which is characterized by a long-range order [60,61]. In particular, one mesogenic dendrimer with shape-persistent morphology has been recently observed to adsorb Xe in the mesogenic and solid states [42], which may increase the applicability of 2D shape-persistent dendrimers in the area of sensing gases. Due to their good ordering, this kind of dendrimer should show a good gas-detecting ability. Recently, a small amount of graphene was mixed with a 2D shape-persistent dendrimer, and a significant increase in the adsorption of Li ions in the solid state was discovered [62]. Therefore, a convenient application in the optimization of batteries can be anticipated.

In addition, since 2D shape-persistent dendrimers with bulky peripheral groups or highly twisted linkers or 3D shape-persistent dendrimers generally have void spaces that are accessible to external molecules, they can be applied in adsorbing volatile organic compounds (VOCs), as mentioned previously. It is worth noting that the frameworks of shape-persistent dendrimers are flexible because they are generally stabilized by H bonding or strong dipole–dipole interactions. When more molecules (VOCs, such as pyridine and benzonitrile), are adsorbed into the void spaces of the shape-persistent dendrimers, the mutual interaction between H bonding and dipole–dipole interactions changes, thus allowing more VOCs to enter the pore of dendrimers, as shown in the literature [50,57]. Compared with MOFs, COFs, or COPs, shape-persistent dendrimers have the advantages of easy purification and reprocessing after usage due to their good solubility in organic solvents. Additionally, because of their flexible frameworks, their capacities for adsorbing volatile organic compounds are comparable with those of MOFs, as reported in the literature [50,57], although their void spaces under CO_2_ are generally smaller than those of MOFs.

## 7. Conclusions

**Figure 16:**
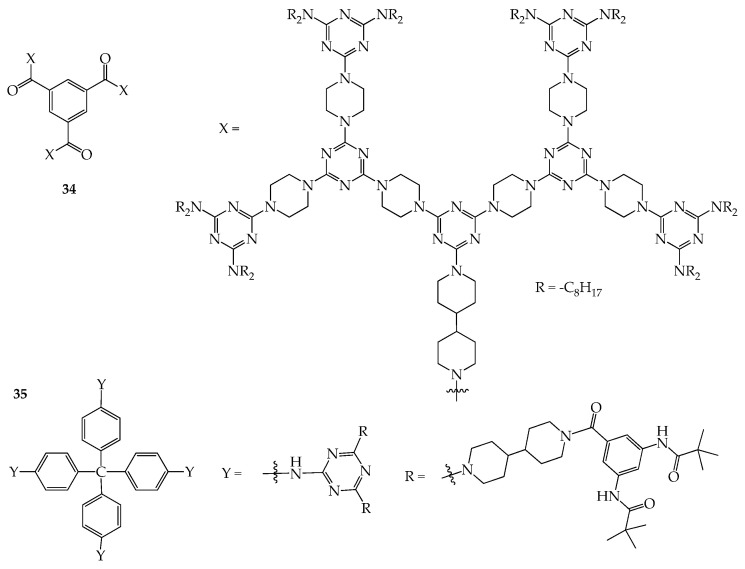
Structures of dendrimers **34** and **35**.

Shape-persistent dendrimers are properly categorized into 2D or 3D molecules in this article, and their corresponding physical properties are briefly described. To allow the void space inside the dendritic frameworks in the solid state to be accessible to external gases, metal ions, or volatile organic compounds and maintain good solubility in organic solvents for the purpose of their application in industry, a 3D central core, suitable rigid linkers, or proper peripheral groups should be incorporated at proper stages of their preparation. Therefore, new shape-persistent dendrimers may be synthesized by varying the corresponding cores, linkers, or peripheral functionalities. In our opinion, a convenient method would be to introduce a different rigid linker into the frame of the dendrimer at certain stages, such as using bipiperidine to replace piperazine in the structures. Because bipiperidine contains a longer chain length than piperazine, this may increase the interstitial gap between the dendritic frame, thus, to some extent, improving the solubility of the dendrimer. This strategy may be applied for preparing 2D or 3D shape-persistent dendrimers, as solubility in organic solvents is a major challenge during their preparation. Once the difficulty of preparation due to their solubility is overcome, other physical attributes and potential uses will be investigated in the future. For example, dendrimer **34** not only maintains a larger intramolecular space arising from the incorporation of biperidine in the central core for adsorbing gases but may also exhibit a mesogenic phase upon thermal treatment. Similarly, dendrimer **35** with a biperidine moiety can also be synthesized, and a larger void space in the solid state may be expected for adsorbing volatile organic compounds (Figure 16). Due to its larger void space, the dendrimer may be used to adsorb environmental contaminants from the aqueous media, which is a new application prospect.

## Data Availability

The data are available on request from the authors.

## Supplementary Materials

The following supporting information can be downloaded at: https://www.mdpi.com/article/10.3390/molecules28145546/s1. Scheme S1: measurement of void space; Table S1: DSC data of dendrimers **1**–**11**. Refs. [47,63,64,65,66] are cited in Supplementary Materials File.
