# Supplementary material for: Shape-Persistent Dendrimers
Source: Molecules. 2023 Jul 20;28(14):5546. doi: 10.3390/molecules28145546 (PMC10385424; doi:10.3390/molecules28145546)
Supplement: Supplementary file 1 [file molecules-28-05546-s001.zip › molecules-2466085-supplementary.pdf]

molecules

Shape-Persistent Dendrimers

Yao-Chih Lu [1], Roberto Anedda [2,*] and Long-Li Lai [1,*]

[1] Department of Applied Chemistry, National Chi Nan University, Puli 545, Taiwan; yaochihlu@gmail.com
[2] Porto Conte Ricerche Srl, S.P. 55 Porto Conte-Capo Caccia, Km 8,400, Loc. Tramariglio 15, 07041 Alghero, Italy
* Correspondence: anedda@portocontericerche.it (R.A.); lilai@ncnu.edu.tw (L.-L.L.); Tel.: +886-49-2910960 (ext. 4976) (L.-L.L.)

Total number of pages of Supporting Information: 3

1 Scheme in Supporting Information

1 Table in Supporting Information

List of the Supplemental Materials

Scheme S1. Measurement of void space.

Table S1. DSC data of **dendrimers 1-11**.

Scheme S1. Estimation of gas sorption.

Brunauer−Emmett−Teller (BET) analyses were examined with a Micrometrics TriStar II Plus system using nitrogen as the adsorbate at 77 K and carbon dioxide as the adsorbate at 195 K, 273K and 298K. All gases used were of 99.9995% purity.

Estimation of Langmuir surface area:

For each point designated for surface area calculations, the Langmuir transformation is calculated as:

$$\mathrm{L} = \frac{P_{rel}}{N_{ads}} \quad \text{(S1)}$$

Here, L is in units of g/cm^3 STP .

A least-squares fit is performed on the (P_{rel}, L) designated pairs where P_{rel} is the independent variable and L is the dependent variable. The following are calculated:

(a.) Slope (S g/cm^3 STP)

(b.) Y-intercept (Y_{int} g/cm^3 STP)

(c.) Error of the slope (S_{err} g/cm^3 STP)

(d.) Error of the y-intercept (YI_{err} g/cm^3 STP)

(e.) Correlation coefficient

Using the results of the above calculations, the Langmuir surface area can be calculated:

$$SA_{Lan} = \frac{CSA \times N_A}{(22414\ cm^3 STP)\left(\frac{10^{18} nm^2}{m^2}\right) S} \quad \text{(S2)}$$

Here, CSA is analysis gas molecular cross-sectional area (nm^2), NA is Avogadro constant (6.0221429$\times 10^{23}\ mol^{-1}$), and S is Slope (g/cm^3 STP) [1-3].

Estimation of Q_{st}:

A virial-type expression comprising the temperature independent parameters a_i and b_i was employed to calculate the enthalpies of sorption for CO2 (at 273 and 298 K). The data were fitted using the equation:

$$lnP = lnN + {}^{1}/_{T} \sum_{i=0}^{m} a_i N^i + \sum_{i=0}^{n} b_i N^i \quad \text{(S3)}$$

Here, P is the pressure, N is the amount adsorbed (or uptake), T is the temperature, a_i and b_i are

virial coefficients, and *m*, *n* represent the number of coefficients required to adequately describe the isotherms (*m* and *n* were gradually increased until the contribution of extra added *a* and *b* coefficients was deemed to be statistically insignificant towards the overall fit, and the average value of the squared deviations from the experimental values was minimized). The values of the virial coefficients a_0 through a_m were then used to calculate the enthalpy heats of adsorption using the following expression.

$$Q_{st} = -R \sum_{i=0}^{m} a_i N^i \quad \text{(S4)}$$

Q_{st} is the coverage-dependent isosteric heat of sorption and *R* is the universal gas constant. The heat of CO2 sorption in this manuscript is determined by using the excess sorption data in the pressure range from 0-1 atm (273 and 298 K), which is fitted by the virial-equation very well.

Dendrimer 17 was measured using the Micrometrics TriStar II Plus, following the procedures outlined in the operating manual. Prior to measurement, **dendrimer 17** underwent pre-processing, which involved vacuum heating and degassing. Subsequently, the instrument was utilized to measure the void sapce of **dendrimer 17**. The obtained results at 195K, 273K, and 298K are presented in Figure S1. The Langmuir surface area and Q_{st} were calculated using the aforementioned formula, resulting in values of 292.92 m^2g^{-1} and 23.4 $KJmol^{-1}$, respectively [4,5].

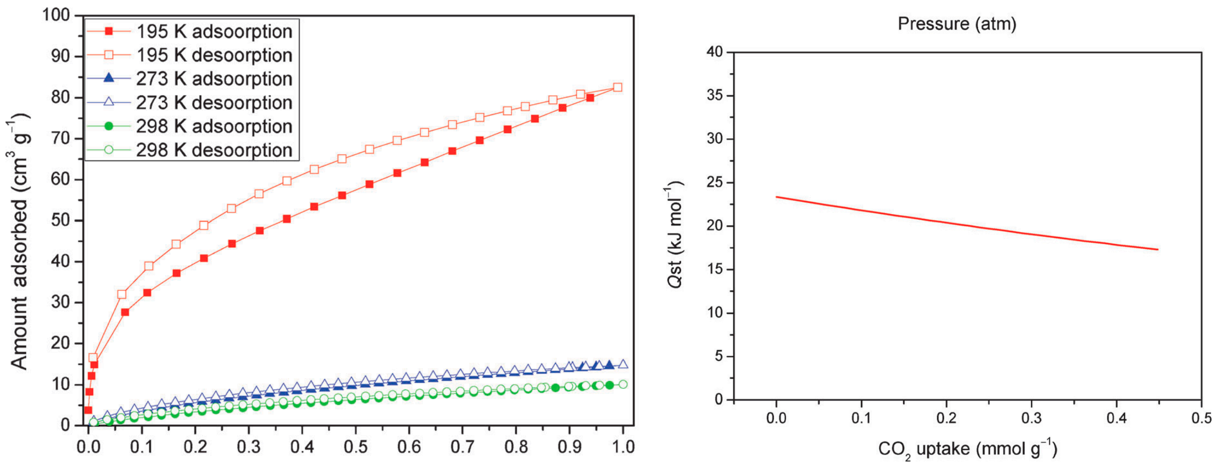

Figure S1. Adsorption properties of **dendrimer 17**. Left: Adsorption isotherms of CO2 at 195 K, 273 K, and 298 K. Right: Isosteric heat (Q_{st}) of CO2 adsorption.

Table S1. DSC data of **dendrimers 1-11**.

**Dendrimer 1**	$\mathrm{Cr} \xrightarrow{101.6} \mathrm{Cr'} \xrightarrow{113.0} \mathrm{Cr''} \xrightarrow{131.9} \mathrm{Cr'''} \xrightarrow{140.5} \mathrm{Col_r} \xrightarrow{172.5} \mathrm{Iso}$	$\mathrm{Iso} \xrightarrow{168.2} \mathrm{Col_r} \xrightarrow{135.6} \mathrm{Cr'''} \xrightarrow{130.4} \mathrm{Cr''} \xrightarrow{95.2,\ 102.8} \mathrm{Cr}$
**Dendrimer 2**	$\mathrm{Cr} \xrightarrow{154.8} \mathrm{Iso}$	$\mathrm{Iso} \xrightarrow{137.0} \mathrm{Col_r} \xrightarrow{100.2} \mathrm{Cr}$
**Dendrimer 3**	$\mathrm{Cr} \xrightarrow{123.4} \mathrm{Col_r} \xrightarrow{144.8} \mathrm{Iso}$	$\mathrm{Iso} \xrightarrow{86.3} \mathrm{Cr}$
**Dendrimer 4**	$\mathrm{Cr} \xrightarrow{82.8} \mathrm{Cr'} \xrightarrow{95.3} \mathrm{Col_h} \xrightarrow{152.3} \mathrm{Iso}$	$\mathrm{Iso} \xrightarrow{147.1} \mathrm{Col_h} \xrightarrow{56.9,\ 63.6} \mathrm{Cr}$
**Dendrimer 5**	$\mathrm{Cr} \xrightarrow{107} \mathrm{Col_h} \xrightarrow{144} \mathrm{Iso}$	$\mathrm{Iso} \xrightarrow{115} \mathrm{Col_h} \xrightarrow{81} \mathrm{Cr}$
**Dendrimer 6**	$\mathrm{Cr} \xrightarrow{109} \mathrm{Col_r} \xrightarrow{168} \mathrm{Iso}$	$\mathrm{Iso} \xrightarrow{146} \mathrm{Col_r} \xrightarrow{87} \mathrm{Cr}$
**Dendrimer 7**	$\mathrm{Cr} \xrightarrow{117.8} \mathrm{Iso}$	$\mathrm{Iso} \xrightarrow{94} \mathrm{Col_h} \xrightarrow{72} \mathrm{Cr}$
**Dendrimer 8**	$\mathrm{Cr} \xrightarrow{111} \mathrm{Col_h} \xrightarrow{123} \mathrm{Iso}$	$\mathrm{Iso} \xrightarrow{115} \mathrm{Col_h} \xrightarrow{94} \mathrm{Cr}$
**Dendrimer 9**	$\mathrm{Cr} \xrightarrow{101.5} \mathrm{Cr'} \xrightarrow{118.0} \mathrm{Iso}$	$\mathrm{Iso} \xrightarrow{114.9} \mathrm{Col_h} \xrightarrow{82.2} \mathrm{Cr}$
**Dendrimer 10**	$\mathrm{Cr} \xrightarrow{88.4} \mathrm{Col_h} \xrightarrow{144.4} \mathrm{Iso}$	$\mathrm{Iso} \xrightarrow{138.4} \mathrm{Col_h} \xrightarrow{68.0} \mathrm{Cr}$
**Dendrimer 11**	$\mathrm{Cr} \xrightarrow{142.6} \mathrm{Col_r} \xrightarrow{172.5} \mathrm{Iso}$	$\mathrm{Iso} \xrightarrow{167.7} \mathrm{Col_r} \xrightarrow{78.9} \mathrm{Cr}$

Cr, Cr′, Cr″ and Cr″″ denote the crystalline phases in various packing.
$\mathrm{Col_h}$ and $\mathrm{Col_r}$ denote hexagonal columnar and rectangular columnar phase, respectively.
Iso denotes isotropic phase.

References

1. Langmuir, I. THE ADSORPTION OF GASES ON PLANE SURFACES OF GLASS, MICA AND PLATINUM. *J. Am. Chem. Soc.* **1918**, *40*, 1361-1403. [CrossRef]
2. Langmuir, I. THE CONSTITUTION AND FUNDAMENTAL PROPERTIES OF SOLIDS AND LIQUIDS. PART I. SOLIDS. *J. Am. Chem. Soc.* **1916**, *38*, 2221-2295. [CrossRef]
3. Langmuir, I. The Evaporation, Condensation and Reflection of Molecules and the Mechanism of Adsorption. *Phys. Rev.* **1916**, *8*, 149-176. [CrossRef]

4. Lee, C.-H.; Tsai, M.-R.; Chang, Y.-T.; Lai, L.-L.; Lu, K.-L.; Cheng, K.-L. Preparation of Unconventional Dendrimers that Contain Rigid NH Triazine Linkages and Peripheral tert-Butyl Moieties for CO2-Selective Adsorption. *Chem. Eur. J.* **2013**, *19*, 10573-10579. [CrossRef]
5. Osterrieth, J.W.M.; Rampersad, J.; Madden, D.; Rampal, N.; Skoric, L.; Connolly, B.; Allendorf, M.D.; Stavila, V.; Snider, J.L.; Ameloot, R., et al. How Reproducible are Surface Areas Calculated from the BET Equation? *Adv. Mater.* **2022**, *34*, 2201502. [CrossRef]
